# Cardiovascular, cortisol and coping responses in urban Africans: the SAPBA study

**DOI:** 10.5830/CVJA-2010-101

**Published:** 2012-02

**Authors:** D Meyburgh, L Malan, JM Van Rooyen, JC Potgieter

**Affiliations:** School for Physiology, Nutrition and Consumer Sciences, North-West University, Potchefstroom, South Africa; School for Physiology, Nutrition and Consumer Sciences, North-West University, Potchefstroom, South Africa; School for Physiology, Nutrition and Consumer Sciences, North-West University, Potchefstroom, South Africa; School for Psychosocial Behavioural Sciences, North-West University, Potchefstroom, South Africa

**Keywords:** cardiovascular, cortisol, coping, responses, Africans

## Abstract

**Objectives:**

To assess the relationships between progression of target-organ damage and cardiovascular, cortisol and coping responses in black urban Africans.

**Methods:**

Urban black African gender groups (*n* = 200) aged 21–62 years from the Sympathetic Activity and Ambulatory Blood Pressure in Africans study were stratified into normotensive and hypertensive groups. Resting and reactivity Finometer blood pressure, fasting sodium fluoride glucose and salivary cortisol values were obtained before and after applying the Stroop and cold pressor tests. Coping strategies were determined and high-resolution ultrasound carotid intima–media scans were done to determine progression of target-organ damage.

**Results:**

A trend of high-normal resting cortisol values during sampling time 1 was demonstrated in all hypertensive men. Both hypertensive gender groups showed increased vascular responses during both mental stressors. During the cold pressor test, vascular responses predicted sub-clinical atherosclerosis in all hypertensive men, independent of sampling time.

**Conclusion:**

Early morning vascular responses in all the hypertensive men could have occurred secondarily to the permissive effect of cortisol on norepinephrine secretion, with subsequent α-adrenergic vasoconstriction. Their α-adrenergic vascular responses during the cold pressor test, however, predicted sub-clinical atherosclerosis, independent of sampling time and cortisol level.

## Abstract

Exposure to chronic stress is a good predictor of future cardiovascular disease (CVD),^1^ and the risk for CVD is mostly increased by an exaggerated blood pressure (BP) response to stress,^1^-^6^ especially in Africans.^7^ Hypertensives have more reactive cardiovascular systems, indicated by greater vascular reactivity, enhanced hypothalamic–pituitary–adrenal axis (HPAA) activity and enhanced salivary cortisol levels when stressed.^3^

Psychological appraisal of stress could contribute to cardiovascular dysregulation through endothelial dysfunction and changes in vascular function.^4^,^5^ Persistent psychosocial stress resulting from urban living in Africans could also lead to increased allostatic load and decreased ability to cope.^6^,^7^ The negative effect that psychosocial stress could have on a person’s health and well-being could, however, be either minimised or exacerbated by his/her choice of coping response.^8^ Coping can be defined as deliberate and effortful attempts to manage situations that we appraise as potentially harmful or stressful.^9^ This includes constantly changing cognitive and behavioural attempts to control the internal and external demands of the situation, which exceed the resources of a person.^10^

Malan *et al*.^11^ revealed that urban Africans indicated behavioural control when using an active coping or problem-solving strategy but contradictorily showed higher cardiovascular risk than rural African men. These findings are opposed to the findings of O’Donnell *et al*., revealing that Caucasian individuals who coped by problem engagement and seeking social support had lower cortisol levels and possibly lower CVD risk.^12^-^14^

Chronic stress can lead to dysregulation of the HPAA with resultant increased circulating adrenocorticotropic hormone (ACTH), cortisol, corticosterone, impaired feedback regulation of the axis and impaired glucocorticoid receptor binding in the hippocampus.^15^ This could indicate the role of glucocorticoids in central control of the cardiovascular system during stress.^16^ Roy *et al*. suggested that resting rather than cortisol stress responses plays a permissive role in sympathetically driven cardiovascular stress responses.^17^

Since stress and related health impairments have become major problems in human life, investigation of the psychobiological pathways that link stress and disease are of major importance in black Africans with a high prevalence of hypertension.^11^,^18^ Therefore, the objective of the study was to assess the relationship between cardiovascular, cortisol and coping responses in urban Africans, as well as its contribution towards progression of target-organ damage (TOD).

## Methods

The SABPA study was a target-population study that included a sample of 200 black African teachers, aged 21 to 62 years, in the Dr Kenneth Kaunda educational district, North-West Province. Hereafter, the black Africans (*n* = 101 men, *n* = 99 women) will be referred to as Africans. Exclusion criteria for the study were: pregnancy, lactation, temperature above 37ºC and users of alpha- and beta-blockers. Participants included had not donated blood or been vaccinated in the previous three months.

Permission to participate was granted by the North-West Education Department and support ensured from the South African Democratic Teachers’ Union. The ethics committee of the North-West University approved the study (00036-07-S6) and the study protocol conforms to the revised ethical guidelines of the Declaration of Helsinki, 2004. A standard subject information sheet was given to the subjects at their screening visit, and an informed consent form was signed prior to the start of the study.

## Experimental procedure

Every morning during the working week, physical activity meters were fitted to four of the 200 teachers between 07:00 and 08:00. Anthropometric measures, i.e. height and weight were taken into consideration for activating the software of the Acticals® activity monitor (Mini Mitter Co, Inc, Bend, OR, Montreal, Canada). The educators then resumed their normal daily activities and were transported at 16:30 to the Metabolic Unit research facility of the North-West University. At 17:00, they were welcomed and the four teachers each received his/her own private bedroom and was familiarised with the experimental setup to lessen anticipation stress.^19^

Pre-counselling for HIV/AIDS was done under supervision by a trained registered nurse. They commenced with the first part of the psychosocial battery at 17:30 under supervision of registered psychologists. A standardised dinner (according to fat, carbohydrate and protein content) was provided at 18:00 and the last part of the psychosocial battery was completed after dinner. Participants were advised to go to bed at 22:00 and instructed to refrain from consuming food, alcohol, caffeine, smoking, exercising and tooth brushing eight hours prior to saliva and blood sampling.^12^

At 05:45 the following morning, the participants were awakened and anthropometric measurements were taken in triplicate by registered anthropometrists according to standardised procedures. We collected blood pressure measures, and saliva and blood samples for cortisol assays from the teachers in semi-Fowlers position in two identical blood pressure stations at two different times (sampling time 1; 06:30–07:00, included teachers 1 and 2, and sampling time 2; 08:30–09:00, included teachers 3 and 4).

Procedures in the two identical blood pressure stations were as follows: blood pressure measurements according to the Rocci/Korotkoff method were taken, followed by resting Finometer blood pressure, and saliva and blood sampling for cortisol determinations. Blood pressure responses were obtained during stressor application while blood sampling was done 10 minutes after stressor exposure. Cortisol saliva responses were sampled 30 minutes after stressor exposure. The same procedure was followed for the second stressor. The two identical blood pressure stations followed exactly the same protocol (± 1.5 hours) under supervision of a registered nurse and doctor. Ultrasound scanning for carotid intima–media thickness (CIMT) followed. Immediate feedback was given on available data and post-counselling regarding HIV status and referrals were made. Thereafter, they received incentives and breakfast, and went back to school.

## Anthropometric measurements

Height was measured with an Invicta Stadiometer (IP 1465, UK) to the nearest 0.1 cm while the participant’s head was in the Frankfort plane. Heels were together, with the buttocks and upper back touching the stadiometer. Mass was measured to the nearest 0.1 kg on a KRUPS scale with the participant wearing minimal clothing and with the weight evenly distributed. Height and mass were used to calculate body mass index (BMI). Waist circumferences (WC) were measured with the participant in a standing position (A & D Company, Japan, Holtain unstretchable flexible 7-mm-wide metal tapes). The WC was taken at the midpoint between the lower costal border and the iliac crest, perpendicular to the long axis of the trunk.

## Blood pressure measurements

Participants were in semi-Fowlers position in the blood pressure stations. After a five-minute rest period, two Riva-Rocci/Korotkoff blood pressure measurements were obtained, with a five-minute rest period between measurements. A suitable cuff size was applied to the non-dominant arm. The second measurement classified participants as hypertensive according to the cutoff points of the European Society of Hypertension Guidelines (Korotkoff sound I: resting SBP ≥ 140 mmHg and/or Korotkoff sound V: resting DBP ≥ 90 mmHg).^20^ Non-invasive continuous beat-to-beat arterial blood pressure recordings were obtained with the Finometer device.^21^ Results were analysed with the Fast Modelflo computer program to provide systolic (SBP) and diastolic blood pressure (DBP), cardiac output (CO), total peripheral resistance (TPR) and arterial compliance (Cw) values.

## Salivary cortisol levels

Resting salivary cortisol levels were obtained 45 minutes after awakening, avoiding the cortisol awakening response (CAR).^12^ Cortisol saliva stressor responses were obtained 30 minutes after exposure to each stressor. Cortisol sampling was done at sampling time 1 (06:30–07:00) and sampling time 2 (08:30–09:00). Optimal hygienic collection of saliva was done with the Salivette® cortisol (Art. No. 51.1534.500) containing a synthetic swab, which was chewed for 45 seconds to one minute and immediately frozen at –80ºC. The cortisol recovery rate in Salivette® cortisol is proven to be almost 100% (Sarstedt AG & Co. Nümbrecht, Germany).

## Stressors

Participants were subjected to mental stressors for one minute in a counterbalanced design, including the Stroop color–word interference task (CWC) and the cold pressor test (CPT), which involved the immersion of the left foot up to the ankle in ice water (4°C).^22^ They received monetary incentive as motivation on completion of the Stroop cards.

## Carotid–intima media thickness (CIMT)

A high-resolution ultrasound carotid intima–media scan^23^ determined target end-organ damage. CIMT images from at least two optimum angles of the left and the right common carotid artery, carotid bulb and internal carotid arterial (ICA) segments were acquired using a Sonosite Micromaxx ultrasound system (SonoSite Inc., Bothell, WA, USA) and a six- to 13-MHz linear array transducer, using the Rudy Meijer protocol.^23^ The images were digitised and imported into the AIMS automated software for dedicated analysis of CIMT. A maximal 10-mm segment with good image quality was chosen for analysis. The program automatically identifies the borders of the CIMT of the near and far wall.

## Questionnaires

The Coping strategy indicator (CSI) of Amirkhan,^24^ which has been successfully used in the South African context, was administered in order to identify participants’ preferred coping strategies. The CSI is a factor analytically derived measure of coping where the following three fundamental coping strategies are revealed: problem solving, seeking social support, and avoidance. Prevalence of smoking and alcohol consumption was determined. Physical activity index (PAI) was determined, where a score of 3 indicated vigorous and 1, indicated low activity.

## Biochemical analysis

A registered nurse sampled fasting blood from brachial vein branches with a winged infusion set, for serum oestrogen as well as sodium fluoride (NaF) glucose levels. Analyses were done with the sequential multiple analyser computer, KonelabTM 20i, Finland. Salivette® cortisol analyses were done with a high-sensitivity enzyme-linked immunosorbant assay (ELISA). The intra- and inter-assay coefficients of variation for cortisol were 7.7 and 9.8%, respectively. The antibody test to indicate positive HIV status was done for each participant, including the First response kit (Premier Medical Corporation LTD, Daman, India) and Confirmatory Pareekshak test (Bhat Bio-tech India (P) LTD, Bangalore, India).

## Statistical analysis

A 2 × 2 analysis of covariance (ANCOVA) adjusted for age, BMI and resting values (blood pressure and cortisol) was conducted to determine interaction between the main effects, i.e. gender and blood pressure status and each of the different variables. Gender groups were stratified according to the ESH guidelines (RivaRocci Korotkoff 2nd measurements) into BP = 120–139/80–89 mmHg and BP ≥ 140/90 mmHg,^20^ hereafter referred to as normotensive and hypertensive African men and women. Coping scores were not normally distributed, therefore median splits stratified participant into low and high responders. Subsequent one-way ANCOVAs followed, adjusted for age, BMI, resting cortisol and blood pressure values.

Multivariate regression analyses were done. Firstly, partial correlations, adjusting for age, BMI and resting values (blood pressure and cortisol), identified independent variables for forward stepwise regression analyses. Secondly, in forward stepwise regression analyses we determined associations in two separate models, i.e. model 1 (for each sampling time) and model 2 (in all hypertensive groups). CIMT was the dependent variable, and independent variables included age, resting and reactivity blood pressure and cortisol values. Multivariate regression values with *p* ≤ 0.05, *r* ≥ 0.35 and adjusted R^2^ 3 0.25 were regarded as significant.

## Results

**Fig. 1:**
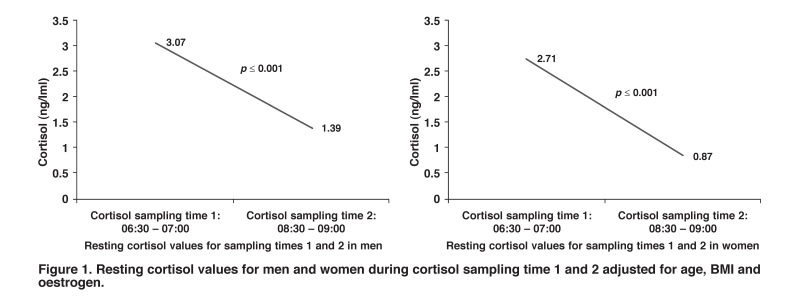
Resting cortisol values for men and women during cortisol sampling time 1 and 2 adjusted for age, BMI and oestrogen.

A single 2 × 2 ANCOVA showed significant interactions on the main effects for low problem solving [df (1, 181) = 4.01, *p* = 0.05] and TPR responses during the CWC test [df (1, 181) = 4.31, *p* = 0.004]. In Fig. 1, the resting cortisol values were significantly higher (*p* = 0.001) during cortisol sampling time 1: 06:00–07:00, than during sampling time 2: 08:30–09:00, in all men and women.

**Figure 2a-d:**
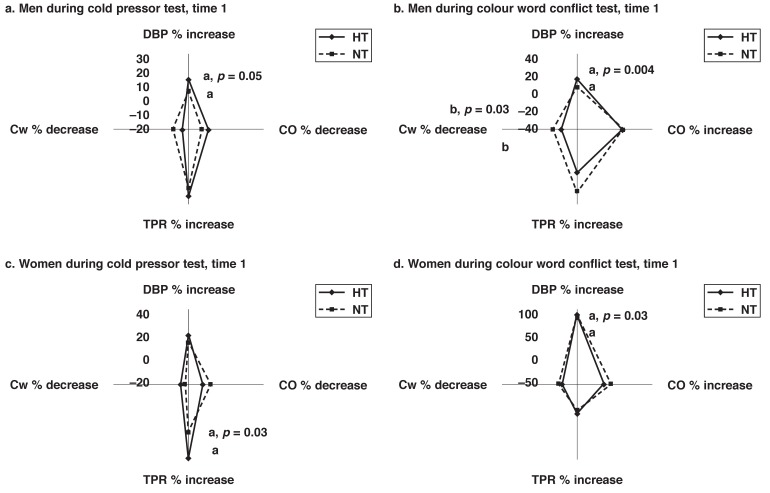
Comparing cardiovascular reactivity values in normotensive (NT) and hypertensive (HT) men (a, b) and women (c, d) independent of age, BMI and resting BP values. DBP, diastolic blood pressure; Cw, arterial Windkessel compliance; CO, cardiac output; TPR, total peripheral resistance.

From Table 1 and Fig. 2, it is clear that hypertensive men were older and showed increased vascular blood pressure during rest (DBP and TPR as well as decreased Cw reactivity) and increased mental stress during sampling time 1 (DBP and decreased Cw reactivity) when compared to their normotensive counterparts. In forward stepwise regression analyses (Table 2), age was associated with progression of sub-clinical atherosclerosis in hypertensive men during sampling time 1. Independent of sampling times though, only vascular responses during the cold pressor test predicted progression of sub-clinical atherosclerosis.

**Table 1:** Descriptive Statistics And Analysis Of Co-Variances, Adjusted For Age And Bmi, Between Normotensive And Hypertensive African Men And Women

	*Men*		*Women*	
	*Normotensive*	*Hypertensive*	*Normotensive*	*Hypertensive*
Participants, *n*	37	64	65	34
Anthropometrics				
Age (years)*	40.9 ± 7.65*	44.5 ± 8.04*	43.7 ± 6.65*	48.6 ± 9.01*
BMI (kg/m^2^)*	26.1 ± 5.12	28.4 ± 6.01	31.1 ± 5.88*	35.9 ± 8.47*
WC (cm)	93.5 (91.7, 95.3)	93.6 (92.3, 95.1)	91.9 (89.9, 93.9)*	96.8 (93.9, 99.6)*
CV parameters				
SBP rest (mmHg)	137 (131, 143)*	152 (147, 156)*	131 (128, 134)*	146 (142, 151)*
DBP rest (mmHg)	81.1 (77.7, 84.5)*	88.6 (86.1, 91.2)*	74.6 (72.8, 76.4)*	82.1 (79.5, 84.6)*
CO rest (l/min)	6.79 (6.20, 7.38)	6.67 (6.22, 7.12)	7.08 (6.67; 7.49)	6.90 (6.32; 7.49)
TPR rest (mmHg/ml/s)	0.97 (0.87, 1.07)*	1.13 (1.05, 1.20)*	0.89 (0.79, 0.99)*	1.09 (0.95, 1.24)*
Cw rest (ml/mmHg)	2.03 (1.93, 2.14)*	1.79 (1.70, 1.87)*	1.94 (1.87, 2.00)*	1.70 (1.61, 1.81)*
Other parameters				
IMT_f_ mean (mm)	0.72 (0.68, 0.77)	0.69 (0.66, 0.73)	0.67 (0.64, 0.71)	0.68 (0.64, 0.72)
Cortisol rest (ng/ml)	2.27 (1.58, 2.96)	2.39 (1.87, 2.91)	1.81 (1.38, 2.22)	1.86 (1.26, 2.46)
Glucose (mmol/l )	5.79 (5.21, 6.39)	5.50 (5.07, 5.93)	5.61 (5.00, 6.23)	5.77 (4.89, 6.64)
Smoking, *n* (%)	10 (27.0)	21 (32.8)	0 (0.00)*	3 (8.82)*
Alcohol usage, *n* (%)	13 (35.1)	28 (43.8)	6 (9.23)	5 (14.7)
Hytension medication (%)	5 (13.5)	10 (15.6)	10 (15.4)*	11 (32.4)*
Diabetes medication (%)	2 (5.41)	1 (1.56)	2 (3.08)	1 (2.94)
HIV, *n* (%)	2 (5.41)*	11 (17.2)*	3 (4.62)	2 (5.88)
PAI low, *n* (%)	30 (81.1)	48 (75.0)	45 (69.2)	24 (70.6)
Coping strategies				
High problem solving	31.2 (30.2, 32.3)	31.1 (30.3, 32.1)	31.2 (30.3, 32.1)	31.1 (29.7, 32.2)
Low problem solving	23.1 (21.5, 24.8)**	25.0 (23.6, 26.5)**	23.9 (21.7, 26.1)	22.2 (19.6, 24.8)
High avoidance	24.8 (23.1, 26.5)	23.4 (21.9, 24.9)	23.2 (21.5, 24.9)	24.3 (22.3, 26.3)
Low avoidance	16.5 (15.2, 17.8)	17.3 (16.3, 18.4)	17.8 (16.5, 19.1)	18.4 (16.8, 20.1)
High social support	28.9 (28.0, 29.8)	29.5 (28.7, 30.2)	29.0 (28.1, 30.1)	28.1 (26.5, 29.5)
Low social support	20.4 (18.3, 22.6)	21.5 (20.0, 22.9)	20.6 (18.9, 22.3)	20.3 (18.4, 22.3)

Values are given with 95% confidence interval. *Values are given according to mean ± standard deviation. *n*, number per group; BMI, body mass index; WC, waist circumference; SBP, systolic blood pressure; DBP, diastolic blood pressure; CO, cardiac output; TPR, total peripheral resistance; Cw, Windkessel compliance; IMT_f_, intima media thickness of far wall; HIV, human immune deficiency virus; PAI, physical activity index. Means with the same superscript letter differ significantly, when *p* ≤ 0.05, when in italic, *p* ≤ 0.09.

**Table 2:** Forward Stepwise Regression Analyses Predicting The Relationship Between Sub-Clinical Atherosclerosis (Cimt) And Cardiovascular Responses In Hypertensive African Men

*Carotid intima–media thickness*		
	*Model 1: hypertensive men, time 1 (n = 32)*	*Model 2: all hypertensive men (n = 60)*
Adjusted *R*^2^	0.61	0.32
	β (± 95% CI)	β (± 95% CI)
Age	0.62 (0.35, 0.89)*	0.55 (0.33, 0.77)*
DBP%, CPT		1.04 (0.16, 1.92)^†^

β denotes standardised regression coefficient and degrees of freedom to enter model, 1.5. Analyses adjusted for resting blood pressure values, where: *p ≤ 0.01, ^†^*p* ≤ 0.05.

In Table 1 and Fig. 2, similar blood pressure trends to that found in hypertensive men were found in hypertensive women, namely they were older with increased vascular blood pressure during rest (DBP, TPR and lower Cw) and increased mental stress (DBP and TPR) compared to their normotensive counterparts in time 1. Additionally, hypertensive women showed more central obesity (WC: *p* = 0.01) compared to their normotensive counterparts. No significant associations for hypertensive women existed to predict progression of sub-clinical atherosclerosis.

## Discussion

The aim of the study was to assess the relationship between cardiovascular, cortisol and coping responses in urban Africans. More men than women were hypertensive (63, 34%, respectively) with no differences in their CIMT levels. As their CIMT levels did not exceed 0.9 mm, which is the cut-off point for diagnosed sub-clinical atherosclerosis, we only indicated trends in the progression of sub-clinical atherosclerosis.^25^

From Fig. 2 it is clear that hypertensive men and women showed increased vascular responses when subjected to acute mental stress. Malan *et al*. demonstrated that a shift from central to more vascular blood pressure responses could be due to uncontrollability of psychosocial stress experienced by Africans.^11^ This may imply that Africans feel overwhelmed with the present-day problems they face. These findings strengthen the idea of more peripheral cardiovascular reactions to stress in hypertensive Africans.^11^,^26^ In support, Nykliček *et al*. added that hypertensives have been found to have a more reactive cardiovascular system than normotensives, and that they show a greater mean DBP reactivity when stressed.^3^ Therefore, this study strengthens these findings and those of Malan *et al*., indicating that Africans exhibit peripheral resistance responses at rest and when exposed to stressful situations.^11^11,^27^

Glucose and salivary cortisol levels in both the normotensive and hypertensive men and women were high-normal (International Diabetes Federation, 5.6 mmol/l; 0.76–2.94ng/ml,^28^ respectively). HPAA hypo-activity seems to be likely in humans experiencing chronic stress, but in hypertensive Africans, their increased resting high-normal cortisol levels could also be due to receptor down-regulation in the hippocampus.^29^,^30^ The resting high-normal cortisol values in particularly the men could impair negative feedback to the hippocampus as a result of receptor down-regulation and, therefore, an experiencing of chronic stress and/or emotional exhaustion is revealed.^29^

Results of urine analyses for cortisol, collected over eight to 24 hours and indicating chronic stress^6^ are, however, not available yet and this is a limitation of this sub-study. Resting cortisol values in both men and women were significantly lower during sampling time 2 compared to sampling time 1. Our data confirm the circadian rhythm that cortisol follows on the observed cortisol concentration at certain times during the day.^31^

Both hypertensive men and women demonstrated higher levels of stress, as indicated by high-normal resting cortisol and glucose values, as well as increased vascular responsiveness during mental stress in cortisol sampling time 1. It was also apparent that in men and women, independent of blood pressure status, their high-normal blood glucose levels, which were above 4.9 mmol/l, could imply increased cardiovascular risk.^32^

In developing countries where cultural disruptions are apparent, such as experienced by black South Africans, this could increase stress and therefore accentuate their CVD risk. This could especially hold true in black Africans with an inherent predisposition to increased salt sensitivity and sympathetic nervous system hyperactivity.^11^,^26^ Findings from other studies^29^,^33^ indicated a synergistic effect of vascular mechanisms and norepinephrine on cortisol, which may further impact on depression or distress via the HPPA.^33^ This indicates that stress and possible HPAA hypo-activity could be important factors in particularly African men’s cardiovascular health, as their vascular responses predicted progression of sub-clinical atherosclerosis.

The mechanism proposed to be active in the hypertensive men is as follows: the resting high-normal cortisol and glucose levels and vascular responses due to acute mental stress could be enhanced via the permissive effect of cortisol on the catecholamines and subsequently vasoconstriction, inhibiting insulin production and vasodilating responses.^34^-^36^ The increased α-adrenergic responses in hypertensive African men could enhance progression towards sub-clinical atherosclerosis as well as the risk for CVD.^37^,^38^ More research in larger sample groups is clearly needed to confirm these speculations.

A typical α-adrenergic vascular pattern was seen in both genders during stressor application, also suggesting a more avoidance/passive coping pattern.^37^ Alpha-adrenergic responses are evoked when a person experiences little or no control during a stressor and is indicative of surrender and feelings of helplessness.^7^ These findings could further indicate that Africans feel overwhelmed and therefore unable to find appropriate solutions for the problems they face. However, none of the coping styles as indicated by the normotensive and hypertensive men and women contributed to the progression of end-organ damage.

Limitations and weaknesses of this study included groups that were too small when different coping strategies were considered within the existing blood pressure stratification. Small groups could have caused the psychological aspect of this study to lose its strength. An additional stress-related hormone such as norepinephrine could have clarified the mechanistic approach. Strengths of the study included the well-controlled psychophysiological design of the study as well as the novelty of data on salivary cortisol levels and associated vascular responses in black South Africans.

## Conclusion

Early morning vascular responses^39^,^40^ in all the hypertensive men could occur secondarily to the permissive effect of high-normal resting cortisol levels on norepinephrine secretion, with subsequent α-adrenergic vasoconstriction. Their enhanced α-adrenergic vascular responses during the cold pressor test predicted sub-clinical atherosclerosis, independent of sampling time and cortisol.
